# The role of RNA binding proteins in cancer biology: A focus on FMRP

**DOI:** 10.1016/j.gendis.2024.101493

**Published:** 2024-12-21

**Authors:** Yunlu Jia, Ruyin Jia, Yongxia Chen, Xuanyi Lin, Nadire Aishan, Han li, Linbo Wang, Xiaochen Zhang, Jian Ruan

**Affiliations:** a Department of Medical Oncology, The First Affiliated Hospital, Zhejiang University School of Medicine, Hangzhou, Zhejiang 310003, China; b The Second School of Clinical Medicine of Zhejiang Chinese Medical University, Hangzhou, Zhejiang 310053, China; c Department of Surgical Oncology, Sir Run Run Shaw Hospital, Zhejiang University School of Medicine, Hangzhou, Zhejiang 310020, China; d Metabolic Hepatobiliary and Pancreatic Diseases Key Laboratory of Luzhou City, The Affiliated Hospital of Southwest Medical University, Southwest Medical University, Luzhou, Sichuan 646000, China

**Keywords:** Cancer immunotherapy, Cancer progression, Cancer stem cells, Fragile X mental retardation protein (FMRP), RNA-binding protein

## Abstract

RNA-binding proteins (RBPs) act as crucial regulators of gene expression within cells, exerting precise control over processes such as RNA splicing, transport, localization, stability, and translation through their specific binding to RNA molecules. The diversity and complexity of RBPs are particularly significant in cancer biology, as they directly impact a multitude of RNA metabolic events closely associated with tumor initiation and progression. The fragile X mental retardation protein (FMRP), as a member of the RBP family, is central to the neurodevelopmental disorder fragile X syndrome and increasingly recognized in the modulation of cancer biology through its influence on RNA metabolism. The protein's versatility, stemming from its diverse RNA-binding domains, enables it to govern a wide array of transcript processing events. Modifications in FMRP's expression or localization have been associated with the regulation of mRNAs linked to various processes pertinent to cancer, including tumor proliferation, metastasis, epithelial–mesenchymal transition, cellular senescence, chemotherapy/radiotherapy resistance, and immunotherapy evasion. In this review, we emphasize recent findings and analyses that suggest contrasting functions of this protein family in tumorigenesis. Our knowledge of the proteins that are regulated by FMRP is rapidly growing, and this has led to the identification of multiple targets for therapeutic intervention of cancer, some of which have already moved into clinical trials or clinical practice.

## Introduction

RNA-binding proteins (RBPs) serve as versatile players in RNA metabolism, engaging in virtually every phase of the RNA lifecycle from transcription to degradation, rendering their roles indispensable.^1^^,^^2^ Their interactions span across a spectrum of RNA species, from messenger RNA (mRNA) to non-coding RNA (ncRNA), each executing distinct yet interconnected biological functions, thereby showcasing the multifaceted functionalities of RBPs. The identification and characterization of RBPs have played a pivotal role in deepening our understanding of gene expression and its regulation in both healthy and diseased states.^3^ RBPs have risen to prominence as pivotal regulators in the intricate dance of cancer development, exerting their influence through meticulous control of post-transcriptional processes and maintaining the delicate balance of gene expression.^4^^,^^5^ A substantial portion of cancer-linked proteins originate from mRNAs that are meticulously managed by RBPs, which fine-tune both translation efficiency and mRNA lifespan.^6^

In recent years, with mounting evidence linking aberrant functions of RBPs to tumorigenesis, tumor progression, and therapy resistance, the scientific community has developed a keen interest in the role of RBPs within the realm of cancer biology. This review aims to consolidate cutting-edge research findings, focusing on a particular RBP known as FMRP, and exploring its multidimensional involvement in cancer. FMRP, encoded by the FMR1 gene, is a versatile RBP fundamental to neural development and known for its role in fragile X syndrome (FXS).^7^ FMRP's versatility manifests in its multifaceted engagement with mRNA metabolism, from suppressing translation to fine-tuning mRNA stability, transport, splicing, and even RNA editing. In the brain, the absence of FMRP disrupts synaptic plasticity, implicating defects in local protein synthesis, cytoskeleton dynamics, and receptor mobility. Beyond neuroscience, research has implicated that alterations in FMRP's interactions with RNA networks have been intimately linked to tumor development.^8^^,^^9^ The aberrant regulation of FMRP directly impacts pivotal processes in cancer progression, including tumor growth, metastasis, epithelial–mesenchymal transition, cellular plasticity shifts, and programmed cell death. Specifically, individuals with FXS, characterized by FMRP deficiencies, demonstrate a lowered cancer incidence, suggesting a protective effect.^10^^,^^11^ The review will delve into the mechanisms by which FMRP exhibits oncogenic or tumor-suppressive functions contingent upon the cellular context, and discuss its potential as a diagnostic biomarker and therapeutic target in cancer.

By examining FMRP's dual role in cancer biology, this review seeks to illuminate its complex interplay with RNA metabolism and its implications for cancer progression and treatment. To achieve this goal, the review is structured around several pivotal sections. Firstly, we will provide an overview of the structural and functional characteristics of RBPs, with a particular emphasis on the distinctive attributes that set FMRPs apart. Subsequently, we will scrutinize the role of FMRP across various cancer types, examining its association with tumor aggressiveness and patient prognosis. Following this, an analysis of FMRP's impact on cancer therapy resistance will be presented, specifically within the contexts of chemotherapy, radiation therapy, and immunotherapy. Lastly, the review will conclude by looking forward to future directions, encompassing the development of targeted interventions against FMRP. This includes leveraging FMRP's dual role in cancer to innovate diagnostic tools and therapeutic strategies designed to enhance patient treatment outcomes.

In light of the growing recognition of the complex roles played by RBPs, particularly the FMRP gene, in cancer biology, this review endeavors to compile the existing knowledge regarding the multifaceted functions of RBPs and FMRP, and their implications for cancer progression and treatment. It will also highlight emerging trends and points of contention within the field. The logical sequence of the review proceeds from the molecular structures and functions of RBPs, through their roles in tumor biology, to a focused presentation of FMRP's structural and functional aspects and its clinical relevance in cancer therapy. This is intended to furnish readers with a comprehensive understanding of FMRP's significance. Ultimately, this review aspires to stimulate further inquiry into the field, guiding the development of innovative strategies to combat cancer. This endeavor is grounded in the mastery of the complexities of RNA metabolism and the pivotal roles played by RBPs, notably FMRP, in this process, paving the way for advancements in precision medicine.

## Overview of RNA-binding proteins

### Classification and characteristics of RBPs

RBPs constitute a versatile ensemble of multifunctional proteins that orchestrate a myriad of fundamental biological processes. Their roles span a wide spectrum, including but not limited to, cell trafficking, spatial localization, developmental programs, cell differentiation, and metabolic pathways. RBPs serve as master regulators that ensure the proper handling and fate of RNA molecules throughout their lifecycle, from synthesis to degradation.^12^ RBPs possess a high degree of spatial and temporal regulatory dynamics within the cell, playing a pivotal role in preserving transcriptome stability and governing the processing and trafficking of RNA. They are engaged in a multitude of processes, including RNA splicing, polyadenylation, stabilization of mRNA, localization of mRNA, and translation.^13^ RBPs exert their regulatory functions through interactions with both coding and non-coding RNAs, such as microRNAs (miRNAs), transfer RNAs, and small interfering RNAs (siRNAs), as well as through partnerships with other proteins.^14^ Through their profound influence on transcript fate and function, RBPs are indispensable architects in sculpting the cellular proteome, thereby dictating the dynamic landscape of cellular physiology.

RBPs can be broadly classified into two categories: conventional and unconventional RBPs.^1^^,^^15^ The distinction is primarily based on whether these proteins possess recognized RNA-binding domains (RBDs), specialized structural elements that facilitate direct contact with RNA molecules.^16^ Conventional RBPs typically feature one or multiple RBDs, including the RNA recognition motif, which is the most thoroughly investigated domain.^17^ Comprising around 80 to 90 amino acids, RNA recognition motifs adopt a conformation featuring two alpha helices and four antiparallel beta strands, enabling them to recognize sequences of 2–8 nucleotides in single-stranded RNA. Another prominent RBD is the K homology (KH) domain, approximately 70 amino acids in length, with a distinctive topology that includes a conserved Gly-X-X-Gly motif for binding to single-stranded RNA regions.^18^ Additional RBDs encompass the double-stranded RBD, cold shock domain, arginine-glycine-glycine (RGG) motifs rich in arginine-glycine-glycine, tyrosine-rich domains, and zinc-finger structures, such as CCHC, CCCH, and ZZ types. On the other hand, unconventional RBPs lack classical RBDs but retain the ability to engage with RNA, often through alternative mechanisms.^19^ Their interactions might be influenced by factors like concentration, affinity profiles, binding and dissociation rates with RNA substrates, and the presence of auxiliary cofactors.^20^

### Post-translational modifications of RBPs

Post-translational modifications (PTMs) of RBPs are a critical mechanism for regulating their functions and activities.^21^^,^^22^ These modifications can impact RBPs at multiple levels, including their localization, stability, and interaction capabilities with RNA and other proteins, as well as their biological activities. Common types of PTMs for RBPs include phosphorylation, acetylation, methylation, ubiquitination, SUMOylation, and N6-methyladenosine (m^6^A) modification. These PTMs are dynamic and can change in response to various cellular signals and environmental cues, allowing for rapid adjustment of RBP function and activity.

Phosphorylation is one of the most common PTMs for RBPs.^23^^,^^24^ Actually, phosphorylation can dramatically alter the activity, localization, and interaction partners of RBPs, influencing their roles in RNA processing, stability, localization, and translation. The reversible nature of phosphorylation, facilitated by phosphatases that remove the phosphate group, allows for dynamic regulation of RBP functions in response to various cellular signals and stimuli. Heterogeneous nuclear ribonucleoproteins A1 and A2 (hnRNP A1/A2) are key members of the RBP family, playing significant roles in cellular stress responses and tumor progression. The phosphorylation of hnRNP A1/A2 can alter its binding characteristics with RNA, influencing RNA splicing and stability.^25^ Under specific conditions, such as the formation of stress granules or changes in the tumor microenvironment,^26^ the phosphorylation of hnRNP A1/A2 promotes the expression of extracellular matrix-related genes.^27^ This could accelerate cell migration and invasion, thereby impacting tumor progression. Human antigen R (HuR) is another RBP that specifically associates with AU-rich elements found in the 3′ untranslated regions (3′ UTRs) of many mRNAs.^28^ The phosphorylation status of HuR may influence its interaction with the mRNAs of apoptosis-related genes such as p53 and Bcl-2, thereby modulating the process of cell apoptosis.^29^^,^^30^ Additionally, members of the SR protein family are RBPs involved in RNA splicing, and their phosphorylation status is crucial for alternative splicing events.^31^ The phosphorylation of SR proteins can enhance their interactions with other components of the splicing machinery, thereby influencing exon selection and splicing patterns.^32^ These examples illustrate how the phosphorylation of RBPs alters their interactions with RNA and regulates RNA metabolism, impacting a variety of cellular processes including cell migration, apoptosis, translational efficiency, and splicing patterns.

Acetylation is another significant PTM experienced by RBPs. p53 binding protein 1 (53BP1) is an RBP associated with DNA damage response, and its acetylation status influences its role in DNA damage repair.^33^ During DNA damage, the acetylation of 53BP1 facilitates its binding to sites of DNA damage, thereby participating in the DNA repair process.^34^^,^^35^ Sam68, an RBP containing polyadenylation signals, can have its RNA binding capability enhanced by acetylation, which subsequently regulates RNA processing and stability.^36^ In certain cancers, abnormal acetylation of Sam68 correlates with tumor development and progression.

Ubiquitination is primarily carried out by three classes of enzymes working in concert: ubiquitin-activating enzymes (E1), ubiquitin-conjugating enzymes (E2), and ubiquitin ligases (E3). This modification process has profound effects on the stability, localization, function, and degradation pathways of RBPs. TAR DNA-binding protein 43 (TDP-43) is an RBP associated with neurodegenerative diseases. Abnormal ubiquitination of TDP-43 is linked to protein aggregation and neuronal cell death, which is particularly evident in diseases such as amyotrophic lateral sclerosis and frontotemporal lobar degeneration.^37^^,^^38^ YTH N6-methyladenosine RNA binding protein 2 (YTHDF2) is an RBP that binds to RNA modified by m^6^A. The ubiquitination and subsequent degradation of YTHDF2 can regulate its interaction with m^6^A-modified RNA, thereby impacting the degradation and translational efficiency of RNA.^39^^,^^40^

PTMs exert profound influences on RBPs by regulating their stability, localization, and functionality, thereby having far-reaching impacts on RNA metabolism and the control of gene expression. These modifications are also associated with various disease states. Understanding the distinct molecular mechanisms of PTMs in RBPs is crucial for unraveling the molecular underpinnings of these diseases and for the development of targeted therapeutic strategies.

## Roles of RBPs in cancer biology: beyond the tip of the iceberg

The RBPs family proteins interact with RNA not only within the nucleus, influencing transcription, but also play roles in regulating other key aspects of cellular biology and genome stability. Dysregulation of RBPs is associated with various cancers, and the intricate functional networks involving RBPs are progressively being elucidated.^41^^,^^42^ This growing understanding offers the potential for identifying novel biomarkers and therapeutic avenues, highlighting the significance of RBPs in disease diagnosis and treatment strategies.

RBPs play a crucial role in the progression of cancer by modulating the expression levels of target RNAs associated with various oncogenic phenotypes such as proliferation, apoptosis, angiogenesis, senescence, and epithelial–mesenchymal transition/invasion/metastasis. Due to their central role in cancer development, RBPs have emerged as potential therapeutic targets in the field of cancer therapy. For instance, Lin28 A/B, an RBP that is up-regulated in multiple cancer types, regulates miRNA processing and affects the maturation of let-7a, thereby promoting tumor growth and metastasis.^43^ Another example is SRSF1, which enhances epithelial–mesen-chymal transition and cell migration in breast and colon cancers by generating ΔRon variants.^44^^,^^45^ Additionally, RBPs like HuR and TIA1 influence cell apoptosis by modulating the stability of programmed cell death (PDCD4) mRNA.^46^^,^^47^ These specific biological examples illustrate the diverse and complex roles of RBPs in cancer progression. With a deeper understanding of the mechanisms by which RBPs function in cancer, we can hope to develop novel therapeutic approaches targeting these key regulatory factors. A representative list of multifaceted roles of RBPs in the pathogenesis and progression of diverse cancer types is shown in Table 1.

**Table 1:** A representative list of multifaceted roles of RNA-binding proteins in the pathogenesis and progression of diverse cancer types.

RNA-binding proteins	Cancer Types	Function	Clinical Significance	Signaling pathways /Target genes	Refs.
HuR	Breast cancer	Regulate cell proliferation, invasion ability, and cell motility	↑ tumor grade ↑ aggressiveness PR (+) ER (+) HER2(+) ↓ survival	G 2/M cell cycle arrest, BRCA1-IRIS, MAPK8, EGFR, miRNA-125, miRNA-16.	48,49
	Gastric cancer		↑ tumor size, NIH risk ↑ Ki67 ↓ DFS.	Snail	50
	Rectal cancer			C2ORF68	51
	Lung cancer		↑ lymph-node involvement ↓ survival	FGFRL1	52
	Gallbladder cancer		↑ advanced tumor stage, histological grade, vascular and perineurial invasion, tumor necrosis, Ki-67 labeling index; ↓ DSS & DFS	HGBC	53
	Pancreatic cancer			PDGFAA	54
LIN28	Breast cancer	CSC-like properties, tumor growth and metastasis	↓ survival	Hippo/YAP1	55
	Hepatocellular carcinoma			IDO1/PD-L1	56
	Colorectal cancer		No association with the time to liver metastasis within 5 years.	SOX2	57
	Gastric cancer		↑Lymph node metastasis ↑advanced TNM stage ↓ prognosis	ZFAS1	58
KHSRP	Colorectal cancer	Promote epithelial cell proliferation, EMT/immune microenvironment/drug resistance		ERRFI1	59
	Lung cancer		↑KHSRP in tumor tissue ↑advanced TNM stage ↑tumor growth	PTEN	60
	Clear cell renal cell carcinoma		↑Advanced clinical stage ↑tumor size, recurrence ↓ prognosis	NEDD4L	61
	Breast cancer		↓prognosis	DEG/RASE	62
ZCCHC4	Hepatocellular carcinoma	Tumor growth	↓ prognosis chemoresistance	AL133467.2	63
La	Lung cancer	Radiotherapy sensitivity/cancer cell proliferation and migration/chemotherapy sensitivity	↓survival	TGFβ	64
	Head and neck cancer		↓ survival	TGFβ	64
	Cervical cancer			CCND1	65
	Head and neck SCC			β-catenin/MMP-2	66
hnRNP A1	Lung cancer	Tumorigenicity, promotion of cancer cell proliferation, metastasis/drug resistance	↓Survival	VRK1	67
	Pancreatic cancer			KRAS-ILK	68
	Breast cancer			SCRIB/ERK signaling pathway	69
	Thyroid cancer			miR-17/miR-18	70
	Gastric cancer			ALOX15	71
	Prostate cancer			ANXA7	72
	Hepatocellular carcinoma			miR-195-5p	73
RBM39/CAPER -α	Acute myeloid leukemia	Cancer cell proliferation and migration	↑drug resistance	PI3K/AKT signaling pathways	74
	Bone marrow malignancy		↓ prognosis	DARS-AS1	75
	Hepatocellular carcinoma			Notch2	76
	Ovarian cancer		↑Resistance to cisplatin and PARP inhibitors	/	77
	Breast cancer		↑Cisplatin resistance	c-Jun	78
RBM38	Colorectal cancer	Inhibiting tumorigenesis/high expression in early liver cancer, low expression in late liver cancer/drug resistance/		PTEN	79
	Hepatocellular carcinoma			p53-mdm2	80
	Gastric cancer		↑ tumor size ↑ depth of invasion ↑ lymph node metastasis ↓ prognosis	/	81
	Lung cancer		↑ CDDP resistance	TRIM17	82
	Breast cancer			TGF-β	83
	Melanoma			A2M/NAMPT/LIF/EBI3/ERAP1	84
PUM1	Gastric cancer	Increased cell proliferation, migration, and colony formation rates	↑Recurrence ↑Metastasis ↓Survival	DEPTOR/PI3K-Akt	85
	Prostate cancer		↓Survival	CDKN1B	86
	Hepatocellular carcinoma			cAMP signaling pathways	87
	Papillary thyroid cancer			MAPK1	88
CIRBP	Breast cancer	Promote proliferation, metastasis, progression/drug resistance		CST3	89
	Hepatocellular carcinomas		↑Risk of recurrence	ROS/CD133	90
	Bladder cancer			HIF-1α	91
	Pancreatic cancer		↓Gemcitabine sensitive	DYRK1B/ERK/p38	92
	Nasopharyngeal cancer			ThermomiR-377-3p	93
RBMS3	Breast cancer	Inhibit cell migration and invasion/drug resistance		PRRX1	94
	Lung cancer		Drug resistance ↓ prognosis	BRAFV600E	95
	Ovarian cancer		Drug resistance	β-catenin/CBP	96
Staufen1	Prostate cancer	Modulates migration and invasion		FOXA1	97
	Rhabdomyosarcomas			HIF2α	98
	Colorectal cancer		↑metastasis	TCEB1	99
	Lung cancer		↑Relapse-free survival	THBS1	100
CELF6	Lung cancer	Promote development/resistance		p53	101
	Colorectal cancer			CD44	102
	Breast cancer		↑Sensitivity to PTX treatment	FBP 1	103
RNPC1	Gastric cancer	Oncogene/tumor suppressor	↑TNM stage	URKB	104
	Lung cancer			CASC2	105
	Breast cancer		↑Doxorubicin resistance	STARD13	106
	Endometrial cancer			MST1/2	107
SERBP1	Glioblastoma	Poor response to chemotherapy and radiotherapy/promote cancer progression	↓survival ↓Chemo/radiotherapy resistance	/	108
	Ovarian cancer			miR-362-3p	109
	Prostatecancer			miR-26a-5p	110
	Hepatocellular carcinoma			circ_0046600	111
	Lung cancer			LINC01468	112

### The role of RBPs in cancer signaling pathways

Studies have demonstrated that RBPs are intricately linked to various cancer-related signaling pathways. One such pathway, the Wnt/β-catenin signaling, holds significant importance in embryonic development and the progression of diverse cancers. When abnormally activated, it provides cancer cells with a means to evade immune checkpoint inhibitors. Notably, RNA-binding motif protein 10 (RBM10), an RBP family member, exhibits tumor–suppressive properties in multiple cancers. RBM10 interacts with β-catenin interacting protein 1 (CTNNBIP1), positively regulating its expression. This interaction disrupts the binding of β-catenin to the transcription factor TCF/LEF, effectively shutting down the Wnt/β-catenin pathway and halting tumor advancement.^113^ In lung adenocarcinoma research, it has been observed that increasing the levels of the RBP PCBP1 slows tumor growth, migration, and invasion. PCBP1 fulfills this tumor-suppressing role by stabilizing DKK1 mRNA and hindering the Wnt/β-catenin signaling pathway.^114^ The Notch pathway is influenced by the RBP musashi 1 (MSI1), which is up-regulated in cancerous cells. MSI1 plays a pivotal role in stem cell and cancer stem cell (CSC) proliferation, operating through both the Wnt and Notch signaling pathways.^115^ Additionally, enabled homolog (ENAH), an actin-binding protein elevated in hepatocellular carcinoma tissues and cells, is linked to a poorer prognosis. ENAH's activity is modulated by the RBP splicing factor 3b subunit 4 (SF3B4), activating Notch signaling and driving tumor progression.^116^ Muscleblind-like splicing regulator 2 (MBNL2), a member of the MBNL family of RBPs, is down-regulated in breast, lung, and liver cancer tissues. Research indicates that MBNL2 controls cancer cell migration and invasion by regulating phosphoinositide 3-kinase (PI3K)/protein kinase B (AKT)-mediated epithelial–mesenchymal transition. In ovarian cancer cells, the RBP insulin-like growth factor 2 mRNA binding protein 2 (IGF2BP2) enhances the malignancy of ovarian cancer by boosting the expression of circular RNAs 0000745 (circ_0000745). This process involves the miR-3187-3p/ERBB4/PI3K/AKT axis.^117^ This mechanism mainly involves miR-3187-3p/(erb-b2 receptor tyrosine kinase 4) ERBB4/PI3K/AKT axis.^118^ Ribosomal protein S7 (RPS7) promotes liver cancer cell adhesion, migration, invasion capabilities, and metastasis. The mechanism is related to the focal adhesion kinase (FAK)/steroid receptor coactivator (SRC) signaling pathway affected by lysyl oxidase-like 2 (LOXL2), a downstream target of RPS7.^119^ RBPs play a crucial role in regulating various cancer-related signaling pathways, including the Wnt/β-catenin and Notch pathways, which significantly impact cancer cell proliferation, migration, invasion, and metastasis.

### Interaction between ncRNAs and RBPs

ncRNAs primarily consist of long ncRNAs (lncRNAs) and miRNAs, which, despite not coding for proteins, exert their effects on gene expression and cellular processes through various mechanisms. The interplay between RBPs and these ncRNAs can influence the progression of cancer.^120^ RBPs can bind to different lncRNAs and promote their export from the nucleus to the cytoplasm, highlighting multiple mechanisms that promote tumor development and progression.^121^

### lncRNAs

lncRNAs such as nuclear paraspeckle assembly transcript 1 (NEAT1), lncRNA H19, and metastasis-associated lung adenocarcinoma transcript 1 (MALAT1) exemplify the complexity of these interactions. NEAT1, overexpressed in multiple cancers, binds to the RBP DEAD-box helicase 5 (DDX5), stabilizing it and activating the Wnt/β-catenin signaling pathway, which is known to promote tumorigenesis.^122^ H19, acting as a miRNA sponge, interacts with RBPs like IGF2BP1, thereby influencing the expression of downstream genes.^123^ MALAT1, interacting with HNRNPC, impacts mRNA stability and plays a role in the regulation of cell cycle and apoptosis, both of which are critical in the context of cancer.^124^ X inactive specific transcript (XIST), another lncRNA, collaborates with RBPs like polycomb repressive complex 2 subunit (SUZ12) and ring finger protein 1 (RING1), engaging in the mechanism of X-chromosome inactivation, which is essential for female embryonic development and has been noted to have implications in certain cancers.^125^^,^^126^ The antisense noncoding RNA in the INK4A locus (ANRIL) gene is linked to the components of the polycomb repressive complex 2 (PRC2), affecting chromatin status and gene expression.^127^^,^^128^ Its abnormal expression has been linked to the risk of cardiovascular diseases and specific cancers, emphasizing its function in epigenetic control and disease vulnerability.

### miRNAs

miRNAs, such as let-7, are central players in post-transcriptional gene silencing. The RBP Lin28 binds to pre-let-7 miRNA precursors, inhibiting their maturation and affecting the expression of let-7 targets, which are often linked to tumor suppression.^43^^,^^57^ Argonaute RISC catalytic component 2 (AGO2), a core component of the RNA-induced silencing complex (RISC), facilitates the binding of miRNAs to their target mRNAs, leading to mRNA degradation or translational repression, a mechanism crucial for the regulation of gene expression in cancer.^129^

### circRNAs

Circular RNA sponge for miR-7 (ciRS-7) contains numerous binding sites for miR-7, serving as competitive endogenous RNAs (ceRNAs) or “sponges” that sequester miR-7, thereby regulating the expression of genes that would otherwise be repressed by miR-7. This interaction between circRNAs and RBPs can influence tumor growth and invasiveness.^130^ Cerebellar degeneration-related protein 1 antisense RNA (CDR1as), a circRNA densely populated with miR-7 binding sites, serves as a potent sponge for this miRNA, intricately governing its cellular function and impacting the pathophysiology of neurodegeneration and oncogenesis.^131^ In contrast, CDR1, a distinct circRNA, engages in a targeted interaction with miR-671, accelerating its decay and thereby sculpting the intricate framework of gene expression control within the cellular milieu.^132^

### snRNAs and snoRNAs

Small nuclear RNAs (snRNAs) and small nucleolar RNAs (snoRNAs) also engage in dynamic relationships with RBPs. Small nuclear ribonucleoprotein polypeptide N (SNRPN), an snRNA, interacts with various RBPs to modulate RNA splicing processes, affecting gene expression patterns in diverse cancers. Small nucleolar RNA, C/D box 116 (SNORD116), an snoRNA, interacts with Drosha, influencing miRNA biogenesis, and although primarily associated with neurodevelopmental disorders such as Prader–Willi and Angelman syndromes, its role in cancer progression is increasingly recognized.^133^

These interactions between RBPs and ncRNAs reveal a rich tapestry of regulatory mechanisms that underpin cancer development, progression, and therapy resistance. By elucidating these complex networks, researchers gain insights into potential therapeutic targets and biomarkers that could revolutionize cancer diagnostics and treatment strategies.

### RBPs as therapeutic targets in cancer treatment

RBPs have indeed shown promise as therapeutic targets in the context of cancer treatment due to their critical roles in various cellular processes that are often dysregulated in malignancies. Drug development strategies targeting RBPs may focus on the RBP itself, its RNA interactions, upstream/downstream alterations in the proteome resulting from changes in RBP function, or any combination of these possibilities (Table 2).

**Table 2:** RNA-binding proteins as molecular targets for cancer therapeutics.

Target	Compounds	Cancer type	Combination regimen	Clinical trial	Chemical structure	*Refs*.
HuR inhibitors	MS-444	Glioblastoma	PARP inhibitors, oxaliplatina, 5-FU, sTRAIL	/		134
	15,16-dihydrotanshinone I (DHTS)	Osteosarcoma	Cisplatin, radiotherapy	/		135
	Triptolide	Liver cancer	Cisplatin, 5-FU, paclitaxel	/		136
	Cryptotanshinone (CT)	Lung cancer/Ovarian cancer	Cisplatin, etoposidea, 5-FU, doxorubicin	/		137
	KH-3	Breast cancer	Docetaxel	/		48
eIF4E inhibitors	Ribavirin	Breast cancer/lymphoma.	Decitabine, brequinar	Phase 1/2		138
	4Ei-1	Breast cancer/lung cancer	Gemcitabine	/		139
	4EGI-1	Breast cancer/nasopharyngeal cancer/multiple myeloma	Radiotherapy	/		140,141
	ISIS 183750	Rectal cancer/prostate cancer	Irinotecan	Phase I/II		142
	Perillyl alcohol	Breast cancer/prostate cancer/pancreatic cancer	/	Phase 2		143
	Cercosporamide	Melanoma/AML	Cytarabine	/		144,145
DEAD/H box RNA helicases inhibitors	Silvestrol	Lymphoma	Rapamycin, doxorubicin, silvestrol	Pre-clinical stage		146
	Pateamine A	Pancreatic cancer	Romidepsin	/		147
	Hippuristanol	Lymphoma/adult T-cell leukemia/multiple myeloma	Dexamethasone	/		148, 149, 150
Musashi proteins (MSI1/MSI2)	Oleic acid	Neuromuscular disease	/	/		151
	Luteolin	Colorectal tumors/tongue squamous cell carcinoma	5-FU	Phase 2		152
	Gossypol	Prostate cancer/NSCLC/glioblastoma/adrenocortical carcinoma/adrenocortical carcinoma.	Docetaxel, cisplatin, rituximab, dalpiciclib and fulvestrant	Phase 2		153
RBM39 inhibitors	E7820	Bone marrow malignancy/solid tumors/colorectal cancer	Irinotecan, FOLFIR,Bevacizumab, Cetuximab	Phase 1/2		154
	E7070	Ovarian cancer/lung cancer/gastrointestinal pancreatic cancer/breast cancer/melanoma	Capecitabine and irinotecan	Phase 1/2		77
LIN28 inhibitors	N-biphenyl or N-dibenzofuran substituen	/		/		155
	C1632	Lung cancer/ovarian cancer/oral squamous cell carcinoma	Metformin	/		156, 157, 158
XPO1 inhibitors	Selinexor	Multiple myeloma/Large B-cell lymphoma/leukemia	/	Phase 3		159
Spliceosome inhibitor	E7107	Acute leukemia/solid tumors/prostate cancer/breast cancer.	/	Phase 1/2		160
PUM1 inhibitors	Morin	Colon cancer/prostate cancer/breast cancer.	Paclitaxel	/		161,162

### Eukaryotic translation initiation factor 4E (eIF4E) inhibitors

Ribavirin is an anti-viral drug that has been repurposed for its potential anti-cancer effects. It acts by inhibiting the cap-binding protein eIF4E, which is crucial for the initiation of cap-dependent translation.^138^ By interfering with this process, ribavirin can inhibit the translation of certain mRNAs that are important for tumor growth. These are synthetic small molecules designed specifically to disrupt the interaction between eIF4E and another protein called eukaryotic translation initiation factor 4 gamma (eIF4G). This interaction is necessary for the assembly of the translational machinery around the mRNA. By blocking this interaction, these inhibitors can prevent the translation of oncogenic mRNAs. Actually, ribavirin is an anti-viral guanosine analog that affects the translation process by targeting eIF4E and has anti-cancer effects on lung, breast, ovarian, and thyroid cancer.^138^^,^^141^^,^^142^ In addition, as a tryptamine phosphoramidate prodrug of 7-benzyl guanosine monophosphate (7Bn-GMP), 4Ei-1 blocks eIF4E cap binding and triggers proteasomal degradation of eIF4E, thereby overcoming gemcitabine resistance in breast and lung cancers.^139^

### HuR inhibitors

MS-444 is a small molecule inhibitor that targets HuR's RNA binding activity. By binding to HuR, it prevents the protein from stabilizing certain mRNAs that promote cell survival and proliferation.^134^ A natural product derivative of Okicenone B (NSC 652240) has been found to bind to HuR and disrupt its function, leading to reduced tumor growth. This compound works by destabilizing mRNAs that are normally stabilized by HuR.^163^ Dehydromutactin, another compound that targets HuR, has been shown to inhibit HuR's ability to bind to specific mRNAs, thereby reducing the expression of genes involved in cell proliferation and survival. MS-444 and 15,16-dihydrotanshinone I are inhibitors of HuR. They inhibit tumor proliferation and progression by targeting HuR and blocking its RNA-binding activity. Another small molecule inhibitor, CMLD-2, inactivates HuR-mediated RNA stabilization. In non-small cell lung cancer and thyroid cancer cells, CMLD-2 treatment reduces the mRNA expression of HuR or competitively binds to HuR, thereby down-regulating target mRNA.^164^ Novel compound KH-3 blocks HuR-RNA binding and may overcome breast cancer resistance to docetaxel,^48^ and inhibit epithelial–mesenchymal transition, migration, and drug resistance of pancreatic cancer cells *in vitro*.

### ASOs and siRNAs

Therapeutic strategies targeting RBPs, including small molecule inhibitors, antagonists, therapeutic peptides, and antisense oligonucleotides (ASOs), are being actively developed and applied in clinical trials. ISIS 183750, an ASO targeting eIF4E, is in phase I/II clinical trials in combination with irinotecan in patients with advanced solid tumor colorectal cancer.^142^ Another ASO, LY2275796, blocks the cap-binding protein eIF4E and has been used in a phase 1 clinical trial in melanoma patients.^165^ Therapeutic peptides are composed of 55 amino acids or fewer; they are easier to synthesize than antibodies and have higher cell permeability and lower immunogenicity. Peptides have the advantages of strong specificity, high selectivity, small size, easy modification, and good biocompatibility. However, translating these findings into effective treatments poses significant challenges, including enhancing drug target specificity to minimize impact on adjacent normal cells, and improving the targeting capability of drug delivery systems to augment therapeutic efficacy.

### Nanoparticles

Nanoparticle formulations for site-of-action delivery and ongoing clinical trials for RBP have emerged as new therapeutic prospects.^166^ The combination of HuR-targeted nanotherapy and AMD3100 produced enhanced suppression of cell growth, migration, and invasion in lung cancer. Specifically, targeting HuR using siRNA-based nanoparticles plus AMD3100 inhibits C-X-C motif chemokine receptor 4 (CXCR4) and suppresses lung cancer metastasis.^167^ Another study evaluated the efficacy of folate receptor-α (FRA)-targeted DOTAP:cholesterol lipid nanoparticles (HuR-FNP) carrying HuR siRNA in suppressing lung cancer cell proliferation and migration. Collectively, HuR-FNP induced G1 phase cell cycle arrest and apoptosis in H1299 cells, leading to significant growth inhibition compared with cells treated with control FNP (C-FNP). The treatment resulted in reduced expression of HuR mRNA and protein, as well as decreased expression of HuR-regulated oncoproteins (cyclin D1, cyclin E, and Bcl-2) and increased expression of the tumor suppressor protein p27 in H1299 cells.^168^ The RBP MSI1 plays a pivotal role in promoting therapeutic resistance in cancer. ASOs and siRNA targeting MSI1 inhibit tumor growth in pancreatic and ovarian cancer *in vivo* and *in vitro*.^169^^,^^170^

Mice with paclitaxel-resistant MDA-MB-231 tumors were resensitized to low-dose paclitaxel by intravenous injection of nanoparticle-loaded eIF4E siRNA.^171^ The upconversion nanoparticle-based delivery system loaded with eIF4E siRNA and Pt(IV) also effectively sensitized laryngeal cancer cells to cisplatin-based chemotherapy and allowed for bioimaging. UCNP@PEI-Pt-PEG@siRNA significantly reduced cell viability and induced apoptosis in Hep-2 cells compared with controls, effectively silenced eIF4E expression, and enhanced the sensitivity of Hep-2 cells to cisplatin, leading to greater cell death and reduced tumor growth. Additionally, upconversion nanoparticles enabled effective *in vitro* and *in vivo* bioimaging, facilitating the monitoring of nanoparticle distribution and therapeutic response.^172^

### Proteolysis targeting chimera

Proteolysis targeting chimeras (PROTACs) are engineered molecules that can trigger the degradation of target proteins by inducing protein degradation through the ubiquitin-proteasome system. Traditional PROTAC consists of three parts: a ligand that recognizes the target protein, a ligand that binds to E3 ligase, and a linker between them.^173^ Traditional PROTACs often fail to pharmacologically treat RBPs. A novel PROTAC capable of functionally targeting RBPs is termed RNA-PROTAC. Overexpression of the RBP Lin28 has been shown to inhibit the biosynthesis of the tumor suppressor miRNA let-7 through direct interaction with pre-let-7.^174^ The establishment of RNA-PROTAC provides a new therapeutic method to solve the problem of RBP-mediated treatment resistance.

## Domain structure of FMRP

In this expansive universe of RBPs, one particular entity, the FMRP gene, emerges as a cornerstone in neurological disorders and a potential player in cancer pathogenesis.

Initially, FMRP garnered attention for its central role in FXS, a genetic condition characterized by intellectual disability and behavioral abnormalities. FMRP, encoded by the FMR1 gene, is a member of the family of RBPs with a predilection for binding to specific RNA sequences, primarily those containing G-quadruplex structures.^175^ This binding capability allows FMRP to modulate the stability, localization, and translation of its target mRNAs, thereby influencing the expression of genes involved in synaptic plasticity, neuronal development, and cognitive function.

However, the story of FMRP does not end with its neurological associations. Intriguingly, accumulating evidence indicates that FMRP may also contribute to the development and progression of various cancers.^9^ Studies suggest that FMRP can act as a tumor suppressor by binding to and stabilizing the mRNAs of genes that inhibit tumor growth. Conversely, in some cancer types, FMRP may adopt a more oncogenic role by regulating the expression of genes that promote cell proliferation and survival. The dual nature of FMRP in cancer is reminiscent of the complex functions of other RBPs in disease, underscoring the intricate interplay between RNA regulation and cancer biology. Thus, as we venture deeper into the uncharted territories of RBPs and their roles in human health and disease, FMRP presents itself as a fascinating case study. Its dual roles in neurological disorders and cancer underscore the need for a more nuanced understanding of RBPs in the context of disease.

**Figure 1:**
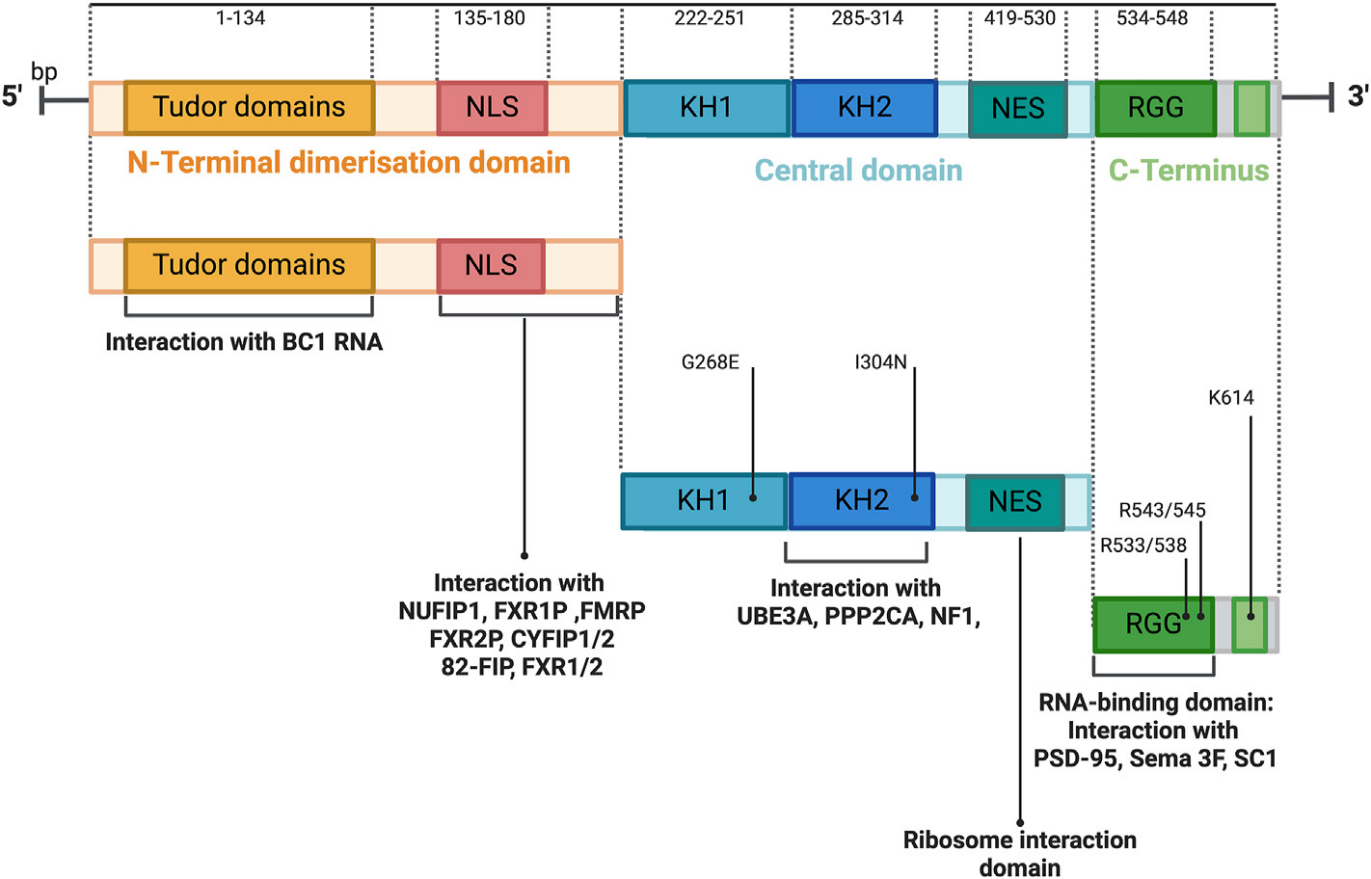
Multi-domain structure of FMRP. Tandem Tudor domains are located at the N-terminus of FMRP; these domains (depicted in yellow) enable specific recognition and binding to proteins with methylated lysine residues, crucial for epigenetic modulation and RNA processing. Central KH domains including KH1 and KH2 (blue) are RNA-binding domains essential for FMRP's function in RNA metabolism and regulation. The C-terminal region of FMRP is rich in arginine-glycine-glycine (RGG) repeats (green), enhancing FMRP's affinity for RNA, particularly G-quadruplex structures. Nuclear localization signals (NLS) (red) and nuclear export signals (NES) (dark green) facilitate FMRP's shuttling between the nucleus and cytoplasm, and localization to the nucleolus, implicating roles in ribosomal biogenesis and mRNA processing.

The domain structure of FMRP is complex and multi-functional, playing critical roles in various cellular processes (Fig. 1). The N-terminal region of FMRP contains two integral tandem Agenet (Tudor) domains, that facilitate recognition and binding to proteins harboring methylated lysine residues, prevalent in histones among others.^176^ These Tudor domains play a vital role in specifically targeting and interacting with proteins where these methylated lysines are present, contributing to various cellular processes including epigenetic regulation and RNA processing. Central to FMRP's function are two important RNA-binding domains known as KH domains, named KH1 and KH2.^177^ These domains are crucial for the protein's role in RNA metabolism and regulation. Both KH1 and KH2 domains in FMRP facilitate binding to RNA molecules. These interactions can be specific to certain RNA sequences or to secondary structures within the RNA. This specificity allows FMRP to regulate the translation of target mRNAs selectively. The C-terminal region of FMRP, which is enriched with RGG repeats, plays a crucial role in the protein's interaction with RNA molecules, particularly enhancing its specificity and affinity for RNA structures such as G-quadruplexes. This domain contributes to the structural flexibility and RNA-binding capability of the proteins that contain it. FMRP also contains nuclear localization signals and nuclear export signals, enabling it to shuttle between the nucleus and cytoplasm and localize to distinct cellular compartments. Nuclear localization signals are short amino acid sequences that direct the protein to the nucleus. FMRP contains one or more nuclear localization signals, which facilitate its import into the nucleus by nuclear import receptors. Once inside the nucleus, FMRP can participate in nuclear processes such as the regulation of mRNA splicing, editing, and nuclear export decisions. The presence of nuclear export signals in FMRP allows it to cycle between the nucleus and cytoplasm, enabling it to carry nuclear-encoded messages and play a role in localized mRNA translation at synapses, which is essential for synaptic plasticity and memory formation. Nuclear localization signals target FMRP to the nucleolus, the center for ribosomal RNA synthesis and ribosome assembly within the nucleus.^178^ The localization of FMRP to the nucleolus suggests roles in ribosomal biogenesis and the regulation of nucleolar processes. This is especially significant in neurons, where rapid protein synthesis is necessary for cell growth and synaptic function.

Key PTMs of FMRP include phosphorylation, ubiquitination, and methylation, which influence its interactions with RNA and other proteins, impacting granule formation, mRNA translation, and ultimately cellular responses to stress. Phosphorylation is the most extensively studied PTM of FMRP. Phosphorylation at residue S499 is crucial for FMRP's regulation in myocyte enhancer factor 2C (MEF2)-induced synapse elimination. Mutations mimicking different phosphorylation states (such as the dephosphorylated mimic S499A and the phosphorylation mimic S499D) impact FMRP's association with RNA granules and its responsiveness to stimuli, thereby modulating the balance between mRNA repression and translation.^179^ Phosphorylation fosters FMRP's coalescence with cell cycle-associated protein 1 (CAPRIN1), a positive regulator of SG assembly and granule-localized protein.^180^ Furthermore, the C-terminal low complexity region (FMRPLCR) of FMRP plays a central role in this process, with phosphorylation augmenting granule formation and potentially inhibiting translation.^181^ The anaphase-promoting complex, an E3 ubiquitin ligase, and its Cdh1 regulatory subunit (Cdh1-APC) can ubiquitinate FMRP, and FMRP ubiquitination through Cdh1-APC opposes SG formation. This suggests that ubiquitination is involved in the intricate regulation of SG formation and function, influencing FMRP dynamics and its role in cellular stress responses. Phosphorylation also plays a critical role in modulating FMRP's function. The phosphorylation of FMRP by casein kinase II (CK2) at specific residues near the RGG box impacts its ability to recognize and bind to RNA.^182^ This modification can influence the selection and regulation of its RNA targets, affecting mRNA metabolism and the cellular response to environmental changes.

FMRP contains intrinsically disordered domains, which are regions lacking a fixed or ordered three-dimensional structure under physiological conditions.^183^ These domains are highly flexible, allowing the protein to adopt multiple conformations and interact with various molecular partners, including RNA and other proteins. FMRP can undergo liquid–liquid phase separation, a process where the protein forms membrane-less droplets or condensates in the cell. Additionally, research has shown that phosphorylation levels in the carboxy-terminal region of FMRP regulate both its phase separation into liquid droplets facilitated by its intrinsically disordered domain and its association with RNA granules involved in translational control.

## FMRP function and RNA biology

**Figure 2:**
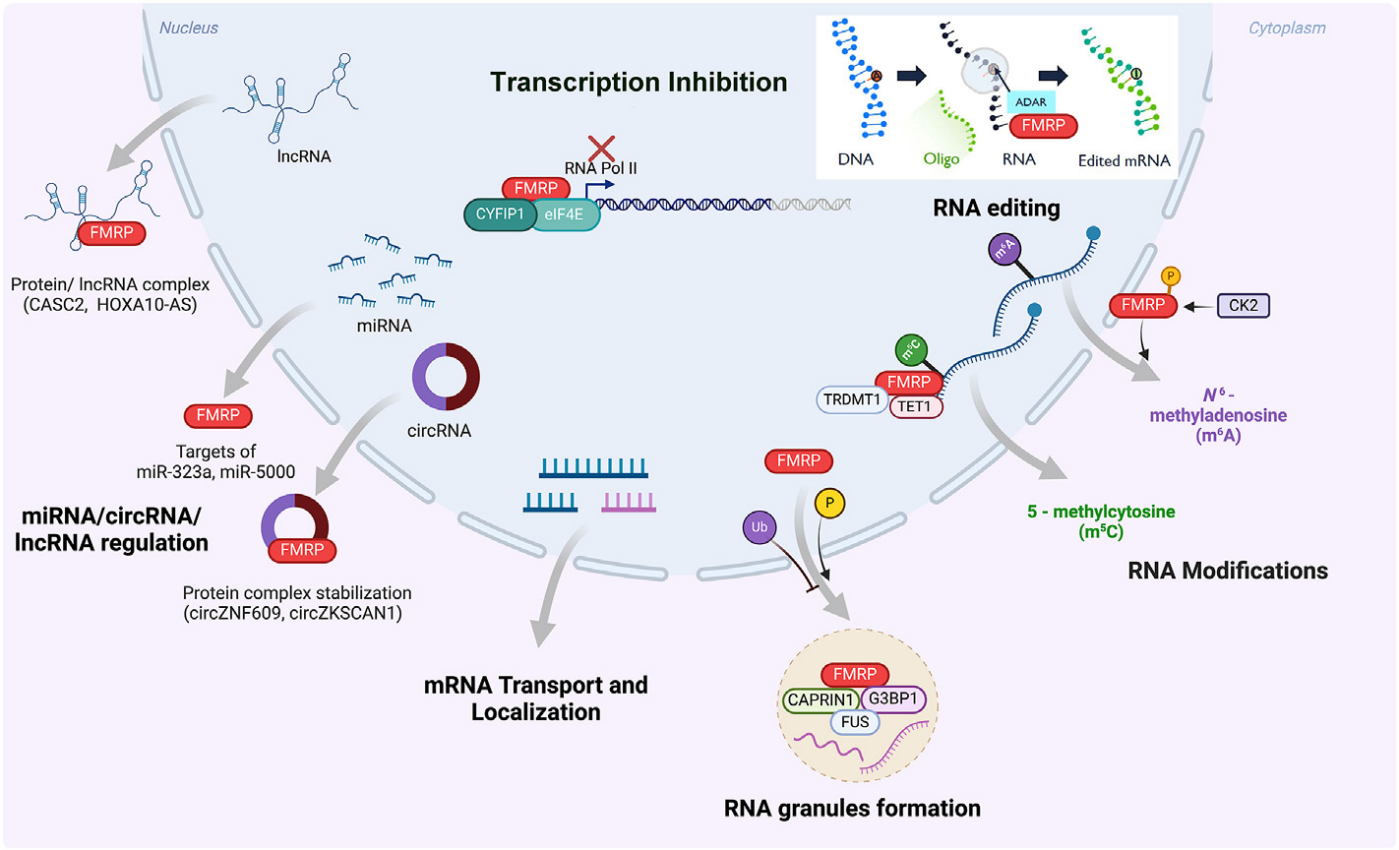
Biological functions of FMRP in RNA biology. The illustrations depict the functional mechanisms of FMRP (depicted in the center) in RNA biology, such as miRNA/cirRNA/lncRNA-mediated regulation, mRNA transport and localization, RNA modification (m^5^C/5-methylcytosine and m^6^A/N6-methyladenosine), RNA editing, and RNA granules formation.

FMRP is renowned for its role as a translational suppressor, which can stall ribosomes on specific mRNAs, particularly those with optimal codons or those encoding synaptic proteins, affecting the translation efficiency. This mechanism involves interaction with ribosomal proteins like the cytoplasmic FMR1 interacting protein 1 (CYFIP1) and potentially blocking elongation factor. FMRP can also directly or indirectly impact alternative splicing by repressing the translation of splicing factors' mRNAs or chromatin-modifying enzymes, which are themselves involved in splicing regulation. Fig. 2 depicts the functional mechanisms of FMRP in RNA biology, including miRNA/cirRNA/lncRNA-mediated regulation, mRNA transport and localization, RNA modification m^5^C (5-methylcytosine) and m^6^A, RNA editing, and RNA granule formation.

### FMRP and mRNA localization

FMRP, an RBP of paramount importance, acts as a master conductor in the intricate symphony of mRNA transport and stability, thereby meticulously orchestrating post-transcriptional gene expression. FMRP binds to mRNAs and escorts them to distant dendrites and axons, highlighting its role in establishing and maintaining neural function and development.^184^ Moreover, FMRP's regulatory influence extends beyond mere transportation; it also wields control over mRNA stability, delicately balancing mRNA decay and preservation. This dual function is facilitated through interactions with the translation machinery and manipulation of mRNA packaging into storage or transport-ready granules.^185^ A recent investigation illuminated FMRP's mechanism in RNA localization by comparing transcriptomes from FMRP-deficient mouse neuronal cells. Researchers revealed a striking correlation: mRNAs that heavily depend on FMRP for neurite transport frequently exhibit abundant G-quadruplex sequences in their 3′ untranslated regions.^185^

### FMRP and RNA modifications

The interactions of FMRP with RNA modifications, particularly m^5^C and m^6^A, highlight its versatile role in cellular processes, including RNA metabolism, DNA damage response, and gene expression regulation. m^5^C modifications in RNA are analogous to methylation in DNA and play critical roles in RNA stability and translation.^186^ FMRP interacts with enzymes responsible for these modifications, notably in the process of demethylation. A key aspect of this interaction is FMRP's facilitation of ten-eleven translocation methylcytosine dioxygenase 1 (TET1), which demethylates m^5^C RNA modifications. FMRP facilitates the demethylation of m^5^C RNA modifications by TET1, particularly following DNA damage.^187^ This mechanism underscores FMRP's role in maintaining genomic stability and its potential impact on cancer development and response to therapy. m^6^A is another crucial RNA modification that regulates various aspects of the RNA life cycle, including stability, translation, and export from the nucleus.^188^ FMRP's interaction with m^6^A-modified RNAs adds another layer to its regulatory functions. While the detailed mechanisms are not fully elucidated, there is evidence suggesting overlap between the mRNAs that FMRP binds (FMRP CLIP targets) and those carrying m^6^A modifications.^189^ This overlap implies that m^6^A modifications might help in the selection of FMRP targets, influencing which mRNAs FMRP interacts with and regulates.

### FMRP regulates RNA editing

Pre-mRNA adenosine deaminase (ADAR) is an RBP that increases transcriptional complexity through a post-transcriptional mechanism called RNA editing.^190^ FMRP was shown to affect RNA editing by interacting with the ADAR proteins in multiple organisms.^191^ Overexpression or down-regulation of drosophila FMRP (dFMRP) has been shown to change the editing efficiency of specific drosophila ADAR (dADAR) targets involved in synaptic transmission.^192^ dFMRP and dADAR interact in the nucleus possibly to regulate RNA editing, and dADAR acts downstream FMRP to modulate synaptic morphology of drosophila neuromuscular junction. FMRP might modulate also ADAR activity by regulating its RNA metabolism. FMRP is associated with ADAR in mice, *D. melanogaster*, and zebrafish, indicating that these interactions serve an important function conserved throughout evolution.^193^ Hints of how FMRP affects RNA editing come from experiments in *D. melanogaster*, in which the expression of an FMRP-ADAR transgene causes editing within the RNA coding sequence. FMRP physically interacts with the enzyme adenosine deaminase acting on RNA 2a (adar2a) in zebrafish, which is part of a family of ADARs responsible for converting adenosine to inosine in RNA, a process known as A-to-I editing.^194^ Notably, increased RNA editing observed in fmr1^−/−^ zebrafish explains the observed hyperlocomotion and enhanced synaptic features, implicating a complex interplay between FMRP, ADAR enzymes, and RNA editing in the regulation of genes that are crucial for neurological function.

### Regulation of RNA granules by FMRP

RNA granules, notably processing bodies (P-bodies) and stress granules (SGs), are essential in mRNA processing, including storage, transport, translation repression, and degradation.^195^ FMRP is a pivotal regulator in the formation and maintenance of these granules. FMRP's role in SG formation was investigated using various experimental models including tumor-derived U2OS cells and immortalized FMRP-knockout mouse embryonic fibroblasts.^196^ However, even in FMRP-deficient cells, SGs were still able to form, suggesting that FMRP is not essential for their initial assembly. It achieves this through RNA binding capabilities and interactions with other granular components. For example, caprin-1, a multifunctional protein involved in various cellular processes, interacts with FMRP via a conserved sequence motif. This, along with G3BP stress granule assembly factor 1 (G3BP1), forms a proposed conserved ribonucleoprotein complex that is potentially hijacked by certain viral proteins, such as the Japanese encephalitis virus core protein, to manipulate cellular machinery.^197^

Several PTMs have been found to regulate SG dynamics, and the most well-characterized PTM in regulating SG dynamics is phosphorylation.^198^ Phosphorylation of FMRP is a key PTM influencing its localization to SGs and granule dynamics. Phosphorylation of FMRP at residue S499 is critical for its regulation in MEF2-induced synapse elimination. Mutations mimicking different phosphorylation states (dephosphomimetic S499A and phosphomimetic S499D) impact FMRP's association with RNA granules and its ability to respond to stimuli, affecting the balance between mRNA repression and translation. This process is interconnected with the activation of metabotropic glutamate receptors and may be influenced by ubiquitination through Cdh1-APC, suggesting a complex regulatory network for SG formation and function.^199^ Specifically, phosphorylation of FMRP was observed to result in its phase separation with CAPRIN1, a protein that positively regulates and localizes to SG assembly. A recent study demonstrated that ubiquitination of FMRP by the E3 ubiquitin ligase Cdh1-APC antagonized the formation of SGs.^180^

FMRP undergoes phase separation, a critical step in granule formation, which is modulated by its phosphorylation status. Phosphorylated FMRP can phase separate with RNA into liquid droplets, which implies that phosphorylated FMRP is more readily able to form translationally repressive granules. The C-terminal low-complexity region (FMRPLCR) is central to this behavior, with phosphorylation enhancing granule formation and potentially repressing translation.^179^ FMRPLCR in forming granules with other RBPs, such as initiation factor 4E-BP2 (eIF4E-BP2), miRNA (miRNA-12b), and decapping protein (Dcp1a), indicating FMRP acts as a hub in granule organization. Conversely, methylation favors granule disassembly and translation. The findings underscore the importance of FMRP's role in controlling mRNA fate through dynamic granule formation and translation via regulated phase separation.

## Roles of FMRP in cancer progression

FMRP's role in cancer biology extends beyond its canonical functions in neurodevelopment and synaptic plasticity, touching upon crucial aspects of cancer progression, such as invasion, metastasis, and drug resistance. Although a significant body of work has illuminated the role of FMRP in FXS, only a handful of studies have produced evidence of its direct or indirect involvement in cancer. Interestingly, some studies suggest that FXS patients might have a decreased risk of developing cancers. However, the exact mechanisms and implications of FMRP levels in cancer are not fully understood. A case study by Kalkunte et al noted reduced glioblastoma invasion in a patient with FXS.^200^ This observation could suggest that the absence or low levels of FMRP might inhibit certain pathways involved in tumor cell invasion and metastasis. In contrast, a high level of FMRP is linked to metastatic breast cancer, and overexpression of the protein in primary breast tumors induces lung metastasis.^201^ The FMRP gene was shown to be a direct target of miR-32323a-3p, with its tumor-suppressive function attributed partly to the repression of FMRP.^202^ Clinically, the analysis of data underscores FMRP's predictive significance across a spectrum of cancers, including clear cell renal cell carcinoma, endometrial cancer, glioma, gastric cancer, and esophageal cancer. Future research should aim to clarify the precise molecular mechanisms by which FMRP levels influence cancer development and progression. Table 3 summarizes the various roles of the FMRP gene in multiple cancer types.

**Table 3:** Roles of FMRP in multiple cancer types.

Cancer type	Expression	Potential translational strategy	Cell lines/Patient tissue/Murine model	Function Pathway/Mechanism of action	References
Colorectal cancer (CRC)	Up	Overcoming programmed cell death resistance	Human epithelial cell lines, patient-derived colon cancer organoids.	Activation of necroptosis through controlling RIPK1 mRNA metabolism	203
Colorectal cancer	Up	Promoting tumorigenesis and metastasis, a target for the prediction and therapy of CRC.	Tissue specimens, human cell lines and BABL/c nude mice	Sustain of EGFR mRNA stability and maintained its expression in an m6A-dependent manner	204
Renal clear cell carcinoma	Down	Negatively associated with the tumor stages; immune-related prognostic biomarker	Bioinformatics approaches	Positively correlated to immune cell infiltration.	205
Clear cell renal cell carcinoma	Up	Promote cancer metastasis	Bioinformatics approaches and human cell lines	Downstream gene of hsa_circ_0037858/miR-5000-3p axis	206
Intrahepatic cholangiocarcinoma	Up	Promote cell invasiveness	Human tissues, human cell lines and male athymic nude mice	Modulating membrane plasticity and invadopodia formation by targeting Cortactin	207
Melanoma	Up	Promote cell migration, invasion and adhesion	Metastatic melanoma tumor tissues and cell lines	Cancer invasiveness-related pathways	208
Melanoma	/	Metastasis of melanoma	Human tumor cell lines, human tissues and BALB/c nude mice	circZNF609 regulates the stability of RAC1 mRNA by combining with FMRP	209
Astrocytoma	Up	Positively related with Ki67 expression and poorer survival	Human tissues and human cell lines	Promote proliferation through activation of MEK/ERK signaling	210
Breast cancer	Up	Correlate with aggressive breast cancer, lung/brain and bone metastases and triple negative breast cancer	Human tissue microarrays, tumor cell lines and Balb/c female mice	FMRP binds hallmarks of EMT (E-cadherin and Vimentin mRNAs) and promote cancer progression.	211,212
Breast cancer	Up	Sensitizes cancer cells to radiation	Human tumor cell lines	Promote mRNA-dependent repair and cell survival in cancer via facilitating TET1-mediated m5C RNA modification demethylation.	187
Prostate cancer	Up	Promote tumor progression	Human cell line and tissue microarrays	Interacts with circRBM33, sustains the mRNA stability of PDHA1, and enhances the ATP production.	213
Prostate adenocarcinoma	Up	Poor prognostic indicator	Bioinformatics approaches	m6A-related cross-talk gene linking prostate adenocarcinoma and periodontitis.	214
Chronic myeloid leukemia	Up	Modify leukemia cells' sensitivity to therapy	Human cell line and tissue	Impacts metabolic adaptation of cells	215
Esophageal cancer	Up	Prognostic indicator	Bioinformatics approaches	m6A-related factors and involved in the immune microenvironment.	216
Esophageal cancer	/	Tumor promoter role	Human cell line and tissue	Target and stabilize HOXA10 mRNA	217
Esophageal cancer	Up	Adverse clinical outcomes	Human cell line and tissue	Activating TLR7-NFκB signaling and upregulating c-Myc level; potential target of miR-323a-3p	202,218
Gastric cancer	Up	Prognosis related	Bioinformatics approaches	m6A-related factors	219,220
Gastric cancer	/	Promote ferroptosis and inhibit GC progression.	Human tissue microarrays, tumor cell lines and Balb/c mice	Downstream gene of LncRNA-CASC2, and activate ferroptosis signaling.	221
Glioblastoma (GBM)	Up	Worse patient outcome	Human tissue microarrays, tumor cell lines and Balb/c mice	Regulation of WNT/β-catenin pathway, promote proliferation of human GBM stem-like cells (GSCs)	222
Glioma cells	Up	Facilitate the proliferation and tumorigenesis of glioma and GSCs	Human tissue microarrays and tumor cell lines	FMR1 maintained circCHAF1A stability, while HOXC8 transcribed FMR1 expression to form a feedback loop, which lead to malignant transformation.	223
Ovarian cancer	Up	Chemotherapy resistance-related	Human OC cell line	Target exosomal miRNA	224
Hepatocellular carcinoma	Up	Accelerate cancer cell metastasis	Human tissue and tumor cell lines	Facilitates IL-6-mediated STAT3 mRNA translation	225
Hepatocellular carcinoma	Up	Reduce the malignant properties of cancer cells	Human tissue and tumor cell lines	Bind circZKSCAN1 and restrain Wnt signaling	226
Urothelial carcinoma	/	Predicting immunotherapy efficacy	Bioinformatics approaches	m6A-related factors	227
Pancreatic ductal adenocarcinoma (PDAC), colon carcinoma, and triple-negative breast cancer (TNBC)	Up	Evade immune destruction.	Human PDAC and colon cancer TMAs, prostate cancer brain metastasis TMA, HCC TMA, human TNBC TMA, genetically engineered mouse models of PDAC, colon cancer, breast cancer, and melanoma.	Remodeling tumor microenvironment, macrophage polarization, and upregulation of the chemokines involved in effector CD8+ T cell recruitment.	9
Pancreatic tumors	/	Invasive growth of cancer cell	Human tumor cell lines and Balb/c mice	Downstream effectors of NMDAR- GKAP axis.	228

### FMRP binds to a variety of RNA targets to regulate cancer biology

By interacting with diverse RNA targets such as G-quadruplex (G4) structures, FMRP modulates the expression of key cancer-related genes, thereby influencing tumorigenesis and progression. RNA G4 structures have been shown to regulate the expression of genes implicated in the hallmarks of cancer, including tumor protein p53 (TP53), vascular endothelial growth factor (VEGF), human telomerase reverse transcriptase (hTERT), and transforming growth factor beta-2 (TGFβ-2).^229^, ^230^, ^231^ Superoxide dismutase 1 (SOD1) is overexpressed in cancers and localized in the cytoplasm.^232^ Through its RGG motif, FMRP recognizes the SoSLIP RNA motif (Sod1 mRNA stem-loops interacting with FMRP) and binds to Sod1 mRNA with a high affinity to activate its translation.^174^ In the context of esophageal cancer, the interplay between FMR1 and lncRNA HOXA cluster antisense RNA 4 (HOXA10-AS), which stabilizes homeobox A10 (HOXA10) mRNA and subsequently regulates the expression of choline dehydrogenase (CHDH), exemplifies the complex layers of post-transcriptional control in cancer cell growth mediated by FMRP.^217^ The discovery of circular RNA ZNF609's (circZNF609) regulatory effect on FMRP-RAC1 mRNA interaction in melanoma presents an intriguing case where modulating RNA stability holds therapeutic promise.^209^ By destabilizing Rac family small GTPase 1 (RAC1) mRNA and suppressing metastasis, this interaction spotlights a novel strategy to combat the aggressive spread of melanoma, a cancer notorious for its invasive properties. FMRP is found to be negatively associated with miR-5000-3p and potentially regulated by the circRNA-miRNA interaction in the malignant metastasis of clear cell renal cell carcinoma.^206^ In hepatocellular carcinoma, FMRP regulates the localization and translation of signal transducer and activator of transcription 3 (STAT3) mRNA and accelerates hepatocellular carcinoma metastasis. FMRP can interact with the 3′ UTR of STAT3 mRNA and promote its localization to protrusions.^225^ The phosphorylation of FMRP at serine 114 emerges as a critical event in this regulatory pathway, as it is essential for enabling IL-6-induced STAT3 translation. This phosphorylation event highlights a potential point of intervention for therapeutic strategies aiming to disrupt the FMRP-STAT3 axis and thereby hinder hepatocellular carcinoma progression. Invasive mucinous adenocarcinoma is a subtype of lung adenocarcinoma with strong invasive ability. Hepatocyte nuclear factor 4 alpha (HNF4α) transcriptionally activates brain cytoplasmic RNA 200 (BC200), which in turn serves as a molecular scaffold, enhancing FMRP's ability to bind to and regulate key cancer-related mRNAs, including that of HNF4α itself.^233^ This creates a self-reinforcing or positive feedback loop, amplifying the oncogenic signals that promote invasive mucinous adenocarcinoma's invasive characteristics. FMRP protein also played a significant role in the progression of colorectal cancer by stabilizing the mRNA of epidermal growth factor receptor (EGFR), thereby enhancing cellular proliferation and migration. This process is dependent on the methylation mark m^6^A. Researchers pinpointed multiple high-confidence m^6^A modification sites in the EGFR mRNA that interact with FMRP.^204^ Of particular interest was a site at position 3758 within the 3′ UTR of EGFR mRNA, where FMRP binding was found to stabilize the mRNA. FMRP binds to specific mRNAs that are involved in epithelial–mesenchymal transition and cellular invasion. Two key mRNAs that FMRP interacts with in this context are E-cadherin and Vimentin.^211^

### FMRP and cancer stem cells

CSCs represent a distinct subpopulation of cells found within tumors that possess unique characteristics resembling those of normal stem cells. These cells are distinguished by their ability to self-renew and differentiate into diverse cell types that make up the heterogeneous tumor mass. CSCs are implicated in tumor initiation, progression, metastasis, therapy resistance, and recurrence.^234^ FMRP has been found to play a significant role in the regulation of CSCs, particularly in glioblastoma^222^ and hepatocellular carcinoma.^226^ The study by Pedini et al provides a detailed investigation into the role of FMRP in glioblastoma, focusing on its modulation of the Wnt signaling pathway and its implications for cancer progression and patient outcomes. Specifically, high levels of FMRP correlate with worse prognosis, while reduced FMRP expression leads to inhibited growth and proliferation of glioblastoma stem-like cells both in cell culture and in mouse models. FMRP appears to regulate glioblastoma stem-like cell proliferation through both canonical WNT/β-catenin and non-canonical WNT-ERK1/2 signaling pathways, influencing the stability of key transcription factors involved in glioma cell malignancy.

Another study elucidates a mechanism where circRNAs can interact with FMRP to negatively regulate CSCs in hepatocellular carcinoma. FMRP is implicated in regulating stemness by modulating a feedback loop with circular RNA ZKSCAN1 (circZKSCAN1), which competes against the FMRP target gene CCAR1 (cell division cycle and apoptosis regulator 1). By physically binding to FMRP, circRNAs prevent it from associating with the CCAR1 complex, which is involved in cell proliferation and survival pathways. This interference reduces the stemness of cancer cells, highlighting an additional layer of complexity in how ncRNAs can influence the behavior of CSCs. The RBP FMRP can also bind to circular RNA CHAF1A (circCHAF1A) and promote its expression by maintaining its stability, while homeobox C8 (HOXC8) also transcribes FMRP expression to form a feedback loop, which may be involved in the malignant transformation of glioma. This novel feedback loop between FMR1, circCHAF1A, miR-211-5p, and HOXC8 in glioblastoma stem-like cells can promote glioma proliferation and tumorigenesis.^223^ lncRNA FMRP-AS1 plays a critical role in maintaining a dynamic equilibrium of cancer stem-like cell populations through the TLR7/NFκB/c-Myc signaling pathway in female patients with esophageal squamous cell carcinoma. FMR1-AS1 exosomes secreted by CSCs of esophageal squamous cell carcinoma facilitate the dissemination of stem-like phenotypes to non-CSCs, influencing tumor heterogeneity and aggressiveness.^218^

### FMRP and programmed cancer cell death

FMRP is involved in the regulation of ferroptosis, a form of programmed cell death characterized by iron-dependent oxidative stress. In gastric cancer, this role is achieved through FMRP's interaction with a lncRNA CASC2 (lncRNA-CASC2), and subsequent modulation of suppressor of cytokine signaling 2 (SOCS2) and suppressor of cytokine signaling (SLC7A11).^221^ Specifically, POU class 6F1, a transcription factor, binds to the promoter region of lncRNA-CASC2, promoting its transcription. The up-regulated lncRNA-CASC2 then interacts with FMRP, stabilizing SOCS2 mRNA and leading to an increase in SOCS2 protein levels. SOCS2, a negative regulator of Janus kinase (JAK)/STAT signaling, suppresses STAT3 activation, which is known to inhibit ferroptosis. Additionally, the lncRNA-CASC2-FMRP complex promotes the ubiquitination and degradation of SLC7A11, a negative regulator of ferroptosis, further facilitating ferroptotic cell death. POU6F1 and lncRNA-CASC2, through FMRP, regulate the SOCS2/SLC7A11 signaling axis to modulate ferroptosis in gastric cancer, suggesting a novel mechanism for controlling cancer stem-like cell survival and potentially targeting cancer stemness in this disease. Necroptosis is an alternative mode of regulated cell death mimicking features of apoptosis and necrosis. In colorectal cancer, FMRP appears to control resistance to programmed cell death, specifically necroptosis, by regulating receptor-interacting protein kinase 1 (RIPK1) mRNA and protein expression, providing insights into the mechanisms underlying cell death resistance in colorectal cancer and suggesting FMRP as a potential therapeutic target for overcoming this resistance.^203^

### FMRP and chemotherapy/radiotherapy resistance

FMRP and TIA1 cytotoxic granule-associated RNA binding protein like 1 (TIAR) exert distinct yet interconnected regulatory roles over protein synthesis, shaping diverse facets of leukemia cell functionality under hypoxic conditions.^215^ Collaboratively, they profoundly impact the nascent proteome within chronic myeloid leukemia cells residing in the bone marrow microenvironment. Specifically, FMRP adjusts the protein synthesis machinery in response to stress, such as hypoxia, enabling chronic myeloid leukemia cells to adapt to their challenging niche – a locale notorious for reduced treatment susceptibility. This adaptive mechanism, targeting the very core of protein translation, emerges as a promising therapeutic intervention point.

In prostate cancer, the narrative unfolds with modified circular RNAs RBM33 (circRBM33) engaging FMR1 (the gene encoding FMRP) to form a complex that preserves pyruvate dehydrogenase E1 subunit alpha 1 (PDHA1) mRNA stability, a critical player in metabolism.^213^ This m^6^A-mediated interaction not only sustains mitochondrial metabolism but also impedes the heightened efficacy of androgen receptor signaling inhibitors, like enzalutamide and darolutamide, suggesting a mechanism by which FMRP contributes to prostate cancer progression and therapeutic resistance. Additionally, FMRP was investigated alongside SGs in head and neck squamous cell carcinoma cells in response to photon irradiation, a form of ionizing radiation used in cancer therapy. Alongside eIF4A3, FMRP is a key constituent of SGs that assemble in radiosensitive cells post-irradiation.^235^ These SG formations may act as cellular bulwarks against radiation-induced damage, influencing the sensitivity of cancer cells to radiation therapy. Unraveling the intricacies of these mechanisms holds the potential to illuminate new avenues for sensitizing radioresistant tumors to therapeutic radiation.

### Cancer-related signaling pathways and FMRP

**Figure 3:**
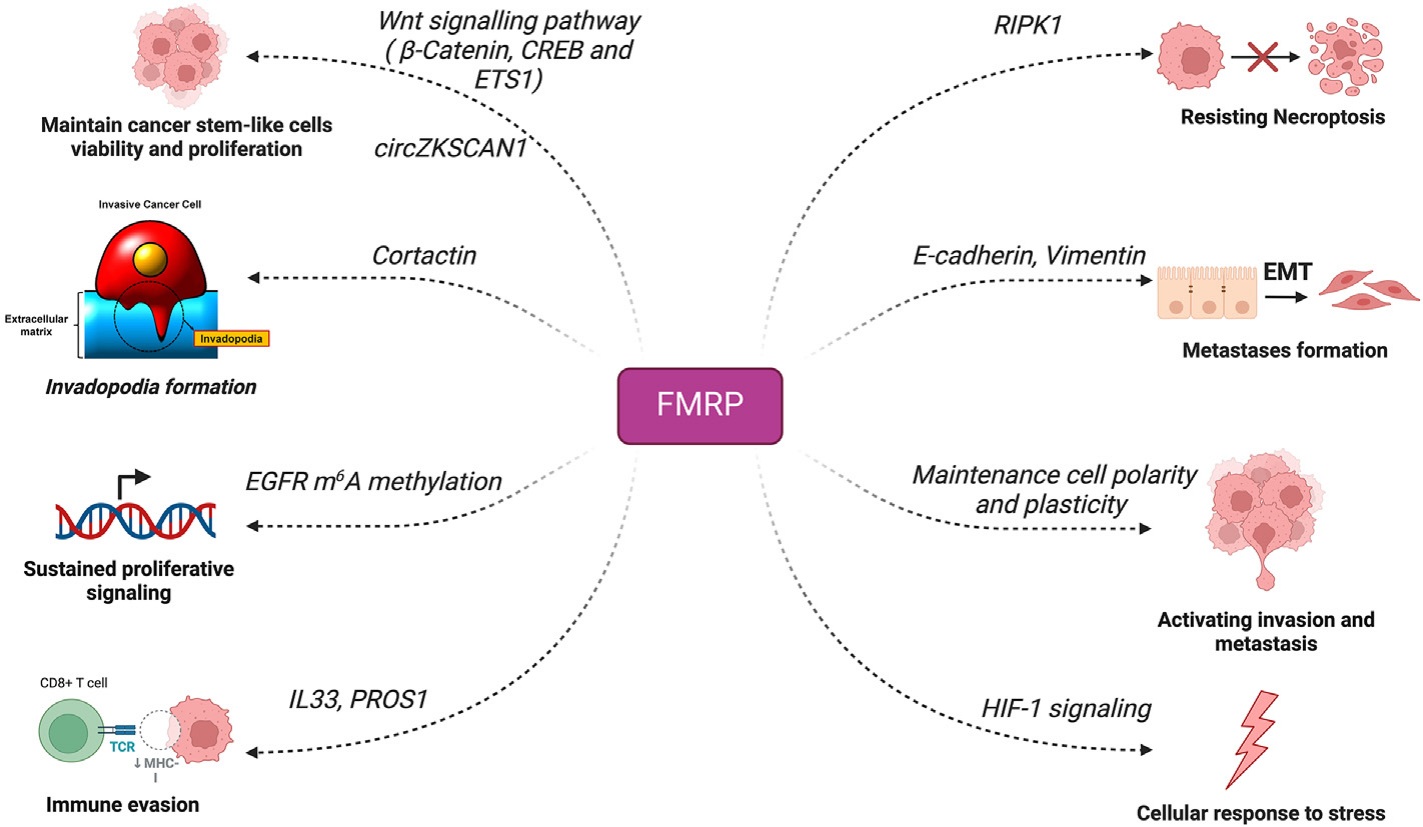
The pivotal role of FMRP in cancer development and progression. High FMRP levels are associated with enhanced invasiveness, metastasis, and drug resistance as well as contributing to the cancer's ability to evade immune surveillance and promote angiogenesis in several cancer types.

FMRP plays a pivotal role in a complex feedback loop that bolsters the production of nerve growth factor, an element vital for both neuronal vitality and function.^236^ This loop assumes heightened significance in the context of cancer, as nerve growth factor not only fosters neuronal expansion but also facilitates tumor progression and the rewiring of the tumor microenvironment.^237^ Elevated nerve growth factor levels stimulate axonal growth and amplify the concentration of cholinergic nerves within the tumor microenvironment, thereby establishing a nurturing environment conducive to cancer cell survival and proliferation. Furthermore, FMRP's involvement in modulating glutamatergic signals via N-methyl-d-aspartate receptors underscores its influence on synaptic transmission — a cornerstone of neuronal communication.^238^ This neural regulatory function hints at FMRP's potential role in cancer-associated mechanisms such as metastasis and invasion, implying that it might facilitate the co-option of neuronal pathways to advance cancer progression. With a focus on gastric cancer, FMRP's impact extends to the activation of cholinergic neuronal transmission, instigating a series of events that ultimately lead to the activation of the Wnt signaling pathway, a well-documented catalyst for carcinogenesis. The stimulation of cholinergic activity via the cholinergic receptor muscarinic 3 (Chrm3) receptor intensifies the discharge of acetylcholine, which, in turn, spurs nerve growth factor production and axon development in gastric stem cells, reinforcing the pro-tumoral niche.^239^ Moreover, FMRP's regulation of ionotropic glutamate receptors, integral to synaptic plasticity, reveals another avenue through which cancer cells can manipulate these mechanisms for invasion and brain infiltration, particularly observed in gliomas and breast cancer brain metastases. In astrocytoma cell lines, enhanced FMRP expression in astrocytoma may promote proliferation through activation of MEK/ERK signaling.^210^ Figure 3 summarizes the pivotal roles of FMRP in cancer development and progression.

### The roles of FMRP in controlling ABC transporters

FMRP has been shown to play a role in controlling ATP-binding cassette (ABC) transporters, which are a family of membrane proteins that transport various molecules across cell membranes. ABC transporters are well-known for their role in multidrug resistance in cancer cells, as they can pump out a wide variety of cytotoxic drugs, thus decreasing the effectiveness of chemotherapy.^240^ A study focusing on the role of FMRP in metabolic regulation found that FMRP negatively regulated the expression of certain proteins involved in lipid metabolism, including ATP binding cassette subfamily A member 1 (ABCA1) and ATP binding cassette subfamily G member 1 (ABCG1).^241^ When FMRP was absent, the expression of these transporters increased, leading to enhanced cholesterol efflux and phospholipid transport. This is consistent with the observation that FMRP-deficient mice exhibit reduced cholesterol levels and increased free fatty acid levels, indicating an up-regulation of lipid metabolism pathways.

The regulation of ABC transporters by FMRP has implications for the development of atherosclerosis. Increased cholesterol efflux facilitated by ABCA1 and ABCG1 helps prevent the formation of foam cells, which are a hallmark of atherosclerosis. By controlling the expression of these transporters, FMRP influences the balance between cholesterol influx and efflux in macrophages. Another study highlighted the interaction between FMRP and the endoplasmic reticulum stress sensor inositol-requiring enzyme 1 (IRE1). Under conditions of endoplasmic reticulum stress, IRE1 activates and phosphorylates FMRP, enhancing its translational suppressor function. This leads to a reduction in the expression of cholesterol transporters like ABCA1 and ABCG1, as well as efferocytosis regulators such as MER proto-oncogene, tyrosine kinase (MerTK), and LDL receptor-related protein 1 (LRP1).^242^^,^^243^ Consequently, cholesterol efflux and the clearance of apoptotic cells by macrophages are suppressed. The implications of this interaction between FMRP and ABC transporters are significant for the development of novel therapeutic strategies aimed at overcoming multidrug resistance in cancer treatment. Understanding the precise mechanisms by which FMRP modulates ABC transporter expression and activity could lead to the identification of new targets for pharmacological intervention.

## Targeting FMRP for cancer immunotherapy

FMRP plays a critical role in modulating the immune gene expression in macrophages, specifically in relation to the balance between pro-inflammatory and anti-inflammatory signals.^244^^,^^245^ By interacting with circular RNAs PPM1F (circPPM1F), FMRP interferes with the translation of PPM1F (protein phosphatase, Mg^2+^/Mn^2+^ dependent 1F), reducing its levels and thereby alleviating its suppressive effect on NF-κB activation. This leads to an enhanced inflammatory response, promoting M1 macrophage activation and exacerbating inflammation in type 1 diabetes mellitus.^246^ A recent study revealed that more innate immune transcripts were up-regulated in resting FMRP-deficient macrophages compared with infected ones, suggesting FMRP might normally repress certain innate immune responses.^247^ These findings collectively propose that FMRP has a role in modulating innate immunity, potentially suppressing unnecessary inflammation. One outstanding work by Zeng et al^9^ reveals a novel mechanism involved in FMRP regulation of immune evasion in cancer for the first time. Authors found high expression of FMRP in most tumors detected using tumor tissue microarrays. Similar results were observed in mouse spontaneous tumors including pancreatic, colon, and breast cancer. Up-regulated FMRP in tumor cells could activate Treg cells by binding to the immune-associated gene mRNA, rejecting and inhibiting CD8 T cell activity. Moreover, protein S (PROS1) ligands and exosomes induce M2-like macrophages to jointly create an immunosuppressive microenvironment. In contrast, tumor cells with low or absent FMRP expression promote the secretion of C–C motif chemokine ligand 5 (CCL5), C-X-C motif chemokine ligand 9 (CXCL9), and C-X-C motif chemokine ligand 10 (CXCL10) from macrophages to create an immune activation microenvironment in cooperation with C–C motif chemokine ligand 7 (CCL7). This suggests that FMRP may play a role in evading immune detection and destruction. However, whether FMRP is involved in the crosstalk between tumor cells and other cells in the tumor microenvironment is not clear.

FMRP also influences the tumor microenvironment by altering the recruitment and activation of various immune cells. By modulating the expression of molecules involved in antigen processing and presentation, FMRP can affect the visibility of cancer cells to the immune system, facilitating immune escape. In melanoma, FMRP has been shown to promote invasion and metastasis, and its silencing reduces the migratory and invasive properties of melanoma cells.^208^ Additionally, it has been implicated in the regulation of immunosuppressive mechanisms within the tumor microenvironment, promoting immune evasion and reducing the responsiveness to immune checkpoint inhibitors. FMRP is a key modulator of the immunosuppressive tumor microenvironment affecting the balance of immune evasion in different tumor models. FMRP has been implicated in a positive feedback mechanism that fosters the production of nerve growth factor, a critical neurotrophin known for its roles in neuronal survival, differentiation, and synaptic plasticity.^248^ In the context of cancer, this interaction takes on new significance as it contributes to tumor progression and the rewiring of the tumor microenvironment. FMRP enters the picture by modulating the effects of glutamatergic signals via N-methyl-d-aspartate receptors, which are associated with cellular processes like metastasis, invasion, and immune suppression.^249^ Gamma-aminobutyric acid (GABA)-ergic signaling is another pathway influenced by FMRP. Normally, FMRP is under the control of glutamate-stimulated N-methyl-d-aspartate receptor signaling in neurons, and interestingly, this signaling pathway is also aberrantly up-regulated in certain cancer cells.^228^ Functional studies have elucidated how GABA/GABAB receptor (GABABR)/β-catenin signaling in cancer cells represses the expression of pro-inflammatory chemokines like C–C Motif chemokine ligand 4/5 (CCL4/5), which attract T cells and CD103^+^ dendritic cells necessary for effective anti-tumor immunity.^250^

**Figure 4:**
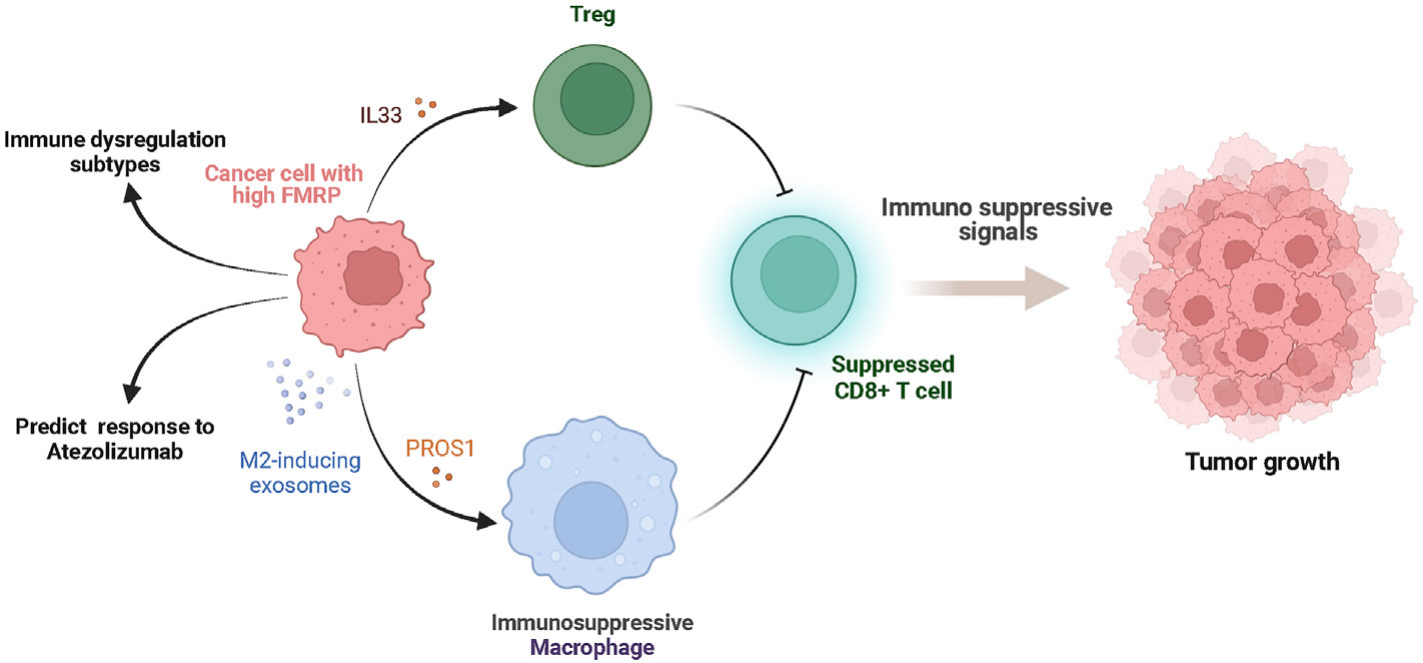
FMRP is a major player in anti-tumor immunity. This figure underscores FMRP's central role in coordinating immune responses against cancer, emphasizing its potential as a key regulator of immune cell function and tumor microenvironment dynamics, and as a promising target for developing novel cancer immunotherapies.

As presented in Figure 4, the involvement of FMRP in tumor immune evasion has significant therapeutic implications. Assessing the expression levels of FMRP in tumors could serve as a biomarker for predicting the response to immunotherapy. Tumors with high FMRP expression might be more adept at evading immune responses, indicating a potential resistance to immune checkpoint inhibitors. For example, the expression of six m^6^A methylation regulators, including IGF2BP1 and FMR1, was significantly different between anti-PDL1 (programmed cell death 1 ligand 1) responders and non-responders. FMRP can individually predict the response to atezolizumab in patients with urothelial cancer.^227^ FMR1 may be a novel immune-related prognostic gene in kidney renal clear cell carcinoma.^205^ Combination therapies that target FMRP alongside traditional immunotherapies could offer a more effective treatment approach.

## Challenges and future directions

FMRP is central to neural development and is primarily known for its role in FXS, a neurodevelopmental condition. FMRP assumes a multifaceted role in both neural system functionality and cancer progression. The connection between FMRP's role in neuroscience and cancer primarily lies in its ability to influence the acquisition of malignant-transforming capabilities by tumor cells, such as invasiveness and immune evasion.^9^ Notably, FMRP participates in a feedback loop that stimulates nerve growth factor production, which is not only vital for neuronal health but also promotes cancer progression and tumor microenvironment remodeling.^251^ By modulating glutamatergic and GABA-ergic signaling, FMRP influences processes such as metastasis, invasion, and immune suppression.^3^ Studies have also revealed that neurons and their projections (axons), as components of the tumor microenvironment, can induce the formation of these cancer hallmarks. Moreover, abnormally activated autocrine and paracrine neural regulatory circuits in cancer cells may constitute a unique “enabling” feature that facilitates the manifestation of hallmark functions and subsequent cancer pathogenesis.^89^ Thus, addressing the challenge of overcoming neuron-specific irreversible damage caused by targeting FMRP requires a nuanced and innovative approach, given FMRP's vital role in both normal neural function and its potential as a therapeutic target in cancer.

FMRP's role in neurons is multifaceted, encompassing the regulation of mRNA transport, stability, and translation, all of which are critical for synaptic plasticity and neural function. Given FMRP's pivotal role in the nervous system, targeting FMRP for cancer therapy must carefully avoid causing irreversible damage to neurons. Such damage could be precipitated by the following scenarios: i) Strategies aimed at reducing FMRP activity in cancer cells might inadvertently impact FMRP's function in neurons, leading to issues with synaptic plasticity and neural health. ii) Disruption of FMRP's control over mRNA stability and translation could adversely affect neuronal survival and function. To minimize neuron-specific injury in FMRP-targeted cancer therapies, researchers are investigating the development of delivery systems designed to confine FMRP-targeting agents to tumor tissues while sparing neural tissues. Besides, strategies that allow for the selective impact on cancer cells while preserving FMRP's normal functions in neurons enable a fine-tuned modulation of FMRP's activity. Addressing the challenge of neuron-specific irreversible damage when targeting FMRP for cancer therapy necessitates a nuanced and innovative approach. The development of targeted delivery systems and precise modulation techniques, coupled with a deeper understanding of FMRP's role in cancer, can pave the way for effective cancer treatments that spare neural function.

The functional role of FMRP exhibits remarkable variations across different types of tumors, reflecting a complexity and context-dependency that allows it to exert both tumor-suppressive and tumor-promoting effects. In glioblastoma, deficiency of FMRP seems to restrain pathways associated with tumor cell invasion and metastasis, suggesting a protective role for FMRP in this type of tumor. In prostate adenocarcinoma, although high levels of FMRP correlate with poor prognosis, its down-regulation might also be linked to disease progression, indicating that FMRP could play a tumor-suppressive role in the early stages of cancer. Conversely, in other types of tumors, FMRP takes on a role that promotes tumor development. In breast cancer, high expression of FMRP is associated with aggressive cancers, including lung, brain, and bone metastases. In hepatocellular carcinoma, FMRP accelerates the metastasis of cancer cells by binding to the 3′ UTR of STAT3 mRNA, enabling its localization to cellular protrusions and the subsequent activation of STAT3 translation. These exemplars elucidate the spectrum of roles that FMRP can adopt across diverse tumor environments, ranging from cancer inhibition to cancer facilitation. The dual nature of FMRP's impact on cancer is influenced by factors such as the specific genetic landscape of the tumor, the surrounding tumor microenvironment, and the particular set of genes whose expression FMRP modulates. Understanding these nuances is crucial for developing targeted therapies that can harness FMRP's tumor-suppressive capabilities while mitigating its tumor-promoting effects.

Current research into the FMRP regulatory network, its interplay with other RBPs, and its impact on epigenetic modifications such as m^6^A methylation, is progressively unraveling the keys to new avenues in precision cancer medicine, particularly in cancer types characterized by FMRP overexpression.^90^ For instance, EGFR is a crucial oncogenic factor that facilitates cell proliferation and migration, with its enhanced activity directly with the progression of tumors. FMRP facilitates colorectal cancer progression by stabilizing the mRNA of EGFR, a process that relies on m^6^A modification, suggesting that targeted intervention at specific m^6^A sites could serve as a therapeutic strategy.^204^ By precisely targeting the mechanisms underlying FMRP-associated mRNA stabilization, future prospects lie in realizing more personalized cancer treatment strategies. Theoretically, by designing small molecule drugs, antibody–drug conjugates, peptide-based drugs, or other nanomedicine techniques that specifically bind to and interfere with the interaction between FMRP and m^6^A-modified sites, the abnormal stabilization of EGFR mRNA can be inhibited, thereby slowing tumor growth and metastasis.^53^^,^^252^^,^^253^ Furthermore, combining this approach with existing immunotherapies, such as anti-PD-1 (programmed cell death protein 1) or anti-PD-L1 treatments, could potentially augment therapeutic efficacy, since aberrant FMRP expression is associated with reduced infiltration of CD8^+^ T cells. This suggests that targeting FMRP to restore immune surveillance represents a promising pan-cancer strategy for treating various malignancies.

Prospectively, a more profound comprehension of FMRP's PTMs and its intricate interplay with other molecules holds the promise to reveal innovative therapeutic targets for cancer intervention. Moreover, given the nuanced relationship between FMRP's functions in neurobiology and its implications in cancer biology, treatments originally conceived for neurological disorders may find themselves with unexpected utility in oncology, particularly within the specialized domain of neuro-oncology. This convergence of neurobiological insight and oncological application heralds a fertile ground for cross-disciplinary innovation, potentially leading to breakthroughs in personalized cancer therapy.

## Funding

This study is supported by the 10.13039/501100001809National Natural Science Foundation of China (No. 82000212), the 10.13039/501100004731Natural Science Foundation of Zhejiang Province, China (No. LQ21H160022), the Medical Health Science and Technology Project of Zhejiang Provincial Health Commission (China) (No. 2024KY989), and Zhejiang Provincial Science and Technology Program for Traditional Chinese Medicine (No. 2025ZR142).

## CRediT authorship contribution statement

**Yunlu Jia:** Methodology, Investigation. **Ruyin Jia:** Investigation. **Yongxia Chen:** Methodology. **Xuanyi Lin:** Writing – review & editing. **Nadire Aishan:** Writing – original draft. **Han li:** Writing – review & editing. **Linbo Wang:** Supervision. **Xiaochen Zhang:** Supervision. **Jian Ruan:** Supervision.

## Data availability

The authors will supply the relevant data in response to reasonable requests.

## Conflict of interests

The authors declared no competing interests.
